# Global restriction of the over-the-counter sale of antimicrobials: does it make sense?

**DOI:** 10.3389/fpubh.2024.1412644

**Published:** 2024-07-03

**Authors:** Carl Llor, Ria Benkő, Lars Bjerrum

**Affiliations:** ^1^University Institute in Primary Care Research Jordi Gol, Barcelona, Spain; ^2^CIBER de Enfermedades Infecciosas, Madrid, Spain; ^3^Research Unit for General Practice, Department of Public Health, University of Southern Denmark, Odense, Denmark; ^4^Department of Clinical Pharmacy, University of Szeged, Szeged, Hungary; ^5^Section and Research Unit of General Practice, Department of Public Health, University of Copenhagen, Copenhagen, Denmark

**Keywords:** drug resistance, microbial, nonprescription drugs, over-the-counter (OTC), pharmacists, health policy

## Introduction

About 80% of antimicrobial agents are used in the community. These antimicrobial agents are either prescribed by healthcare professionals or directly purchased by consumers without a valid prescription, being known as over-the-counter sales of antimicrobials, commonly through sources such as community pharmacies. While many countries prohibit the sale of over-the-counter antibiotics, more than 50% of antibiotics are globally acquired without a prescription (1). Two comprehensive studies have examined the extent of over-the-counter antibacterial requests that lead to the non-prescription supply in community pharmacies on a global scale. In the latest study, the authors reviewed 38 studies from 24 different countries, revealing an overall pooled proportion of non-prescription supply of antibiotics of 62% (2). The issue of over-the-counter antibiotic availability is particularly pronounced in developing countries, such as South America, Africa, and some areas in Asia, where regulations governing the sale and distribution of medicines are either non-existent or inadequately enforced. However, this problem is not confined to these regions alone; even in developed countries, including those in southern Europe, non-prescription antibiotic supply is a concern (3). Furthermore, in affluent countries, antibiotics can also be sold in community pharmacies without a prescription. Surprisingly, various over-the-counter sore throat lozenges commonly used globally contain locally delivered antibiotics (4).

Most patients in middle- and low-income countries who receive antibiotics without a prescription typically show symptoms related to upper and lower respiratory and urinary tract infections. Many of the antibiotics without medical prescription fall into the category of the World Health Organization (WHO) AWaRe category of “Watch antibiotics”, such as macrolides, cephalosporins or quinolones, or “Access antibiotics” with a broad spectrum of action, such as amoxicillin or amoxicillin-clavulanic acid. Interestingly, both types of antibiotics are frequently sold even in cases in which equally effective narrow-spectrum antibiotics could serve as the initial treatment option. Some studies show that fluoroquinolones are commonly recommended and supplied over the counter for uncomplicated urinary tract infections instead of the recommended first-line narrow-spectrum antibiotics, such as trimethoprim, fosfomycin, pivmecillinam or nitrofurantoin.

## Over-the-counter sale of antimicrobials is not always inappropriate

Not all sales of antimicrobials without medical prescriptions should be considered inappropriate or illegal practices. There are situations in which the non-medical initiation of certain antimicrobials may be justified. For infections that can be diagnosed based on symptoms alone—such as acute uncomplicated cystitis, impetigo, tinea corporis, herpes zoster, and bacterial conjunctivitis—or in cases of recurrent infectious problems, such as uncomplicated urinary tract infections or genital herpes, where patients are well-acquainted with the symptoms, pharmacists might be allowed to dispense antimicrobial agents. Pharmacists could be allowed to dispense first-line antimicrobial agents in situations in which simple point-of-care testing can be used, such as in adult women with symptoms of uncomplicated urinary tract infections with a positive urine dipstick result or an acute sore throat with a positive rapid antigen detection test result for streptococcal infection. In a recent Welsh study, community pharmacists using clinical scores and point-of-care testing for patients with suspected streptococcal pharyngitis resulted in fewer antibiotics being dispensed (5). In the UK, pharmacists legally supply azithromycin to patients with a positive chlamydia test as well as their sexual partner(s) (6). Legislative changes in countries like Canada and New Zealand have empowered designated pharmacists to prescribe and dispense antibiotics for specific conditions. For instance, in New Zealand, pharmacists have the authority to prescribe and dispense trimethoprim for the short-term treatment of an uncomplicated urinary tract infection (6). The cases mentioned above should not be considered and categorized as real over-the-counter sales but rather as legal, pharmacist-only dispensing practices.

## Determinants of over-the-counter sales of antibiotics

The roots of over-the-counter sales of antimicrobials are complex, stemming from various factors. To gain a deeper understanding of why this problem persists, qualitative studies are necessary (7). These studies provide insights about the main determinants, with low awareness about antimicrobial resistance (AMR), and the adverse effects of antibiotics among dispensers being among the most common reasons. Moreover, in one interview-based study, the respondents believed that the financial interests of business owners and consumer demand further added to this phenomenon. In many pharmacies, particularly those which are smaller, some pharmacists may be reluctant to refuse customer requests due to concerns about losing loyal clientele. Dispensing of non-prescription antibiotics has been found to be higher in cases in which the patients are known. Studies identify patient complacency as a significant factor motivating the dispensing of antibiotics without a prescription. According to community pharmacists, patients who have previously found antibiotics effective and view them as a solution are more likely to request and obtain these medications. Additionally, community pharmacists sometimes dispense antibiotics based on the understanding that doctors would prescribe antibiotics in clinical situations such as those presented in the pharmacy, mainly in villages and small towns where pharmacists know the prescription habits of the local doctors.

## Association of the over-the-counter sale of antimicrobials with AMR and other deleterious effects

The WHO has recognized AMR as a significant global threat to public health worldwide. The relationship between antibiotic consumption and AMR is clearly established and poses a substantial challenge for low-to-middle-income countries, which have experienced recent increases in antimicrobial consumption. Analyzing pharmaceutical sales data from 2000 to 2010 across 71 countries worldwide, Van Boeckel et al. investigated variations in antibiotic consumption (8). The study revealed significant differences in antibacterial usage, with notably higher rates observed in the BRICS countries—Brazil, Russia, India, China, and South Africa. In these nations, over-the-counter sales contribute significantly to the excessive use of antibacterial agents. Over-the-counter sale of antibiotics in developing countries, outside the member states of the Organization for Economic Co-operation and Development (OECD), is cited as a contributing factor to the higher prevalence of resistance (9).

The unauthorized dispensing of antimicrobials for self-limiting conditions, such as cough resulting from respiratory tract infections, common colds, and sore throat, significantly contributes to the escalation and dissemination of AMR. A study by Wesgate et al. emphasized that the presence of local antibiotics in sore throat lozenges that can be acquired over the counter in the western world poses a risk for the emergence of cross-resistance between pathogens (4).

Non-prescription antimicrobial use is frequently linked to irrational treatment courses and inappropriate choices of drugs and doses. This irrational use of antibiotics can have various consequences apart from AMR, including delayed hospital admissions, and the masking of infectious disease diagnoses. For instance, temporarily suppressing tuberculosis symptoms with fluoroquinolones may lead to delayed diagnosis and result in patients receiving multiple antibiotic courses for a wrong diagnosis (10).

## The impact of strengthening regulations prohibiting over-the-counter sales of antimicrobials

Several prior investigations have demonstrated that the enforcement of restrictive measures leads to substantial reductions in the over-the-counter sales of antimicrobials following policy interventions. This reduction has been observed in numerous studies, mainly carried out in various American, African, and Asian nations, as well as certain southern European countries. The ultimate impact of regulations must be assessed not only regarding the sale of antimicrobial, but also focusing on their outcomes of interest, such as trends in AMR among common pathogens. However, the impact of these stringent policies on AMR is controversial. A global assessment revealed that amoxicillin and clavulanate stood out as the most widely purchased antibiotic across 75 nations, constituting nearly 50% of overall antibiotic sales (11). Even though the WHO considers this to be an Access antibiotic, it has a broad spectrum and is more prone to developing resistance compared to antibiotics with a narrower spectrum. The sale of oral and parenteral antimicrobials with a higher propensity to induce AMR, such as Watch and Reserve Antibiotics, should be prohibited from being dispensed at community pharmacies without medical prescriptions globally unless they are empowered to prescribe and dispense antibiotics for specific conditions. Additionally, administrative, and regulatory measures should be enforced for broad-spectrum antibacterials known to contribute significantly to AMR.

## Some solutions aimed at curbing AMR

As community pharmacies often serve as the initial point of contact for the public and they are the most accessible healthcare facilities worldwide, pharmacist training and competences should be extended toward infectious disease prevention and treatment. Many patients have problems in obtaining access to general practitioners or pediatricians or simply opt to bypass the clinician, driven by factors such as time or travel constraints or lengthy appointment waiting times. In such cases, community pharmacists, trained in antibiotic stewardship, could play a crucial role in facilitating prompt access to antibiotics and promoting their rational use. There is evidence in the literature to confirm that the proximity of a pharmacy decreases the demand for primary care (12). The independent prescriber pharmacist and nurse practitioner training, that was initiated in the UK in 2006 could serve as a good policy example for other countries with limited access to primary care providers (13). These pharmacists must adhere to standard treatment protocols to address specific bacterial infections, provide guidance on the proper use of antibiotics, educate the public about the risks associated with the indiscriminate use of this class of medicines, and deter unnecessary antibiotic use for non-bacterial infections. By treating symptoms appropriately and ensuring patients understand their illness, community pharmacists can contribute to the rational use of antibiotics. Therefore, it is suggested that policies in these settings should prioritize promoting the rational use of antibiotics rather than solely restricting their supply without a medical prescription.

To tackle the issue of indiscriminate antimicrobial use, comprehensive strategies that go beyond administrative or regulatory measures are essential (14). Interventions in developing countries should be tailored to local contexts and address specific root causes. Except for tuberculosis and specific upper airway infections such as otitis media and streptococcal pharyngitis, in which shorter antibiotic courses are less effective, clinical trials have demonstrated that most bacterial respiratory tract infections show equal efficacy with fewer adverse events in shorter compared to standard antibiotic courses (15). This is based on the principle of “shorter is better”, initially conceived one decade ago. In a landmark study, it was discovered that a 3-day antibiotic regimen showed comparable efficacy to an extended 8-day treatment for adults hospitalized due to mild to moderate-severe community-acquired pneumonia. Notably, this equivalence was observed in individuals who demonstrated significant improvement following the initial 3 days of therapy, accounting for nearly 80% of the study participants (16). Despite evidence supporting shorter treatment durations, guidelines often recommend longer courses, leading to unnecessary antibiotic use. As a result, numerous physicians continue to opt for longer courses, often administering 7- and 10-day courses. WHO Access antibiotics, such as penicillin V or amoxicillin, should be promoted as first-line treatments for respiratory infections, with marketing emphasizing 3-day courses. Regulations on over-the-counter antibiotic sales should consider limiting long therapy courses. Overall, the promotion of shorter antibiotic courses not only benefits patients but also mitigates AMR, presenting a crucial step forward in addressing this global health challenge.

## Conclusions

Addressing the indiscriminate use of antimicrobials requires a multifaceted approach involving educational, regulatory, and cultural interventions across both low- and middle-income countries as well as Western nations. We outline some straightforward solutions designed to reduce over-the-counter sales of antimicrobials and combat AMR. While over-the-counter sales are not always detrimental, community pharmacists, trained in antibiotic stewardship, can play a pivotal role in ensuring timely access to antibiotics and promoting their responsible usage. Embracing the principle of shorter courses of WHO Access antibiotics, particularly for respiratory and urinary tract infections, can effectively combat antimicrobial resistance while maintaining safety standards.

## Author contributions

CL: Conceptualization, Formal analysis, Visualization, Writing – original draft. RB: Conceptualization, Formal analysis, Visualization, Writing – original draft. LB: Supervision, Writing – review & editing.

## References

[1] Cars O Nordberg P. Antibiotic resistance. The faceless threat. Int J Risk Saf Med. (2005) 17:103–10. Available online at: https://www.reactgroup.org/uploads/publications/react-publications/antibiotic-resistance-the-faceless-threat.pdf

[2] Auta A Hadi MA Oga E Adewuyi EO Abdu-Aguye SN Adeloye D. Global access to antibiotics without prescription in community pharmacies: a systematic review and meta-analysis. J Infect. (2019) 78:8–18. 10.1016/j.jinf.2018.07.001 29981773

[3] Guinovart MC Figueras A Llop JC Llor C. Obtaining antibiotics without prescription in Spain in 2014: even easier now than 6 years ago. J Antimicrob Chemother. (2015) 70:1270–1. 10.1093/jac/dku526 25558070

[4] Wesgate R Evangelista C Atkinson R Shepard A Adegoke O Maillard JY. Understanding the risk of emerging bacterial resistance to over the counter antibiotics in topical sore throat medicines. J Appl Microbiol. (2020) 129:916–25. 10.1111/jam.14682 32352619

[5] Mantzourani E Cannings-John R Evans A Ahmed H. To swab or not to swab? Using point-of-care tests to detect Group A Streptococcus infections as part of a Sore Throat Test and Treat service in community pharmacy. J Antimicrob Chemother. (2022) 77:803–6. 10.1093/jac/dkab470 35038341 PMC8865000

[6] International Pharmaceutical Federation (FIP). Fighting Antimicrobial Resistance: The Contribution of Pharmacists. The Hague: International Pharmaceutical Federation (2015). Available online at: https://www.fip.org/file/163 (accessed June 14, 2024).

[7] Roque F Soares S Breitenfeld L López-Durán A Figueiras A Herdeiro MT. Attitudes of community pharmacists to antibiotic dispensing and microbial resistance: a qualitative study in Portugal. Int J Clin Pharm. (2013) 35:417–24. 10.1007/s11096-013-9753-4 23397322

[8] Van Boeckel TP Gandra S Ashok A Caudron Q Grenfell BT Levin SA. Global antibiotic consumption 2000 to 2010: an analysis of national pharmaceutical sales data. Lancet Infect Dis. (2014) 14:742–50. 10.1016/S1473-3099(14)70780-7 25022435

[9] Bryce A Hay AD Lane IF Thornton HV Wootton M Costelloe C. Global prevalence of antibiotic resistance in paediatric urinary tract infections caused by *Escherichia coli* and association with routine use of antibiotics in primary care: systematic review and meta-analysis. BMJ. (2016) 352:i939. 10.1136/bmj.i939 26980184 PMC4793155

[10] Satyanarayana S Kwan A Daniels B Subbaraman R McDowell A Bergkvist S. Use of standardised patients to assess antibiotic dispensing for tuberculosis by pharmacies in urban India: a cross-sectional study. Lancet Infect Dis. (2016) 16:1261–8. 10.1016/S1473-3099(16)30215-8 27568359 PMC5067371

[11] Jacobs TG Robertson J van den Ham HA Iwamoto K Bak Pedersen H Mantel-Teeuwisse AK. Assessing the impact of law enforcement to reduce over-the-counter (OTC) sales of antibiotics in low- and middle-income countries. A systematic literature review. BMC Health Serv Res. (2019) 19:536. 10.1186/s12913-019-4359-8 31366363 PMC6670201

[12] Erku DA Mekuria AB Surur AS Gebresillassie BM. Extent of dispensing prescription-only medications without a prescription in community drug retail outlets in Addis Ababa, Ethiopia: a simulated-patient study. Drug Healthc Patient Saf. (2016) 8:65–70. 10.2147/DHPS.S106948 27471409 PMC4948721

[13] Graham-Clarke E Rushton A Noblet T Marriott J. Non-medical prescribing in the United Kingdom National Health Service: a systematic policy review. PLoS ONE. (2019) 14:e0214630. 10.1371/journal.pone.0214630 31356615 PMC6663007

[14] Guinovart MC Figueras A Llor C. Selling antimicrobials without prescription - far beyond an administrative problem. Enferm Infecc Microbiol Clin. (2018) 36:290–2. 10.1016/j.eimc.2016.10.006 27866752

[15] Spellberg B. The maturing antibiotic mantra: “shorter is still better”. J Hosp Med. (2018) 13:361–2. 10.12788/jhm.2904 29370317 PMC6483376

[16] el Moussaoui R de Borgie CA van den Broek P Hustinx WN Bresser P van den Berket GE. Effectiveness of discontinuing antibiotic treatment after three days versus eight days in mild to moderate-severe community acquired pneumonia: randomised, double blind study. BMJ. (2006) 332:1355. 10.1136/bmj.332.7554.1355 16763247 PMC1479094

